# Prognostic Significance of Systemic Inflammation Markers in Testicular and Penile Cancer: A Narrative Review of Current Literature

**DOI:** 10.3390/life13030600

**Published:** 2023-02-21

**Authors:** Aleksandar Janicic, Milos Petrovic, Milica Zekovic, Nenad Vasilic, Vesna Coric, Bogomir Milojevic, Marko Zivkovic, Uros Bumbasirevic

**Affiliations:** 1 Clinic of Urology, University Clinical Center of Serbia, 11000 Belgrade, Serbia; 2 Faculty of Medicine, University of Belgrade, 11000 Belgrade, Serbia; 3 Centre of Research Excellence in Nutrition and Metabolism, Institute for Medical Research, National Institute of Republic of Serbia, University of Belgrade, 11000 Belgrade, Serbia; 4 Institute of Medical and Clinical Biochemistry, Faculty of Medicine, University of Belgrade, 11000 Belgrade, Serbia

**Keywords:** inflammation, testicular cancer, penile cancer, prognostic biomarkers

## Abstract

In contemporary clinical practice, biomarkers are indispensable in the assessment and management of oncological patients. Although established serum tumor markers (beta human chorionic gonadotropin (bHCG), alpha fetoprotein (AFP), and lactate dehydrogenase (LDH)) have an indisputably important role in the management of patients with testicular cancer (TC), the application of these tumor markers may be accompanied with certain limitations, implying the need for additional biomarkers. Contrary to TC, there is a lack of established serological biomarkers for penile cancer (PC) and the management of this urological malignancy is based on multiple clinicopathological parameters. Therefore, the identification and rigorous analytical and clinical validation of reliable biomarkers are considered pivotal for improving PC management. Inflammation may be associated with all stages of oncogenesis, from initial neoplastic transformation to angiogenesis, tissue invasion, and metastasis. Accordingly, an array of inflammation-related indices have gained increasing attention as emerging predictors of oncological outcomes. The clinical usefulness of systemic inflammation markers was reported in many urological and non-urological malignancies. The aim of this narrative review is to summarize current scientific data regarding the prognostic and predictive significance of systemic inflammation markers in TC and PC patients.

## 1. Introduction

Biomarkers have a significant role in the assessment and management of oncological patients. In accordance with BEST (Biomarkers, EndpointS, and other Tools; Food and Drug Administration) biomarker categorization, they can be utilized for screening, establishing a diagnosis, staging, estimating prognosis, and identifying disease recurrence [1]. From the 1970s, serum tumor markers, such as beta human chorionic gonadotropin (bHCG), alpha fetoprotein (AFP), and lactate dehydrogenase (LDH), have been implemented in the routine clinical management of testicular cancer (TC) [2]. Although indisputably valuable, the application of these tumor markers may be accompanied with a number of limitations, thus necessitating the need for new candidates [3,4]. Unlike TC, current clinical care of penile cancer (PC) is lacking well-established biomarkers. Appertaining to the contemporary guidelines, the management of this urological entity is contingent on a cluster of diverse clinicopathological parameters [5]. Therefore, the identification, rigorous analytical assessment, and clinical validation of reliable biomarkers are considered pivotal for improving PC management.

The correlation between inflammation and the cancerogenesis has been postulated by Virchow since 1863. The scientific interest in this field has been revived over the past few decades, with a particular focus on potential biomarkers and promising treatment targets. Tumor-infiltrating leukocytes, dysregulated cytokine, growth factor, and chemokine production, as well as tissue remodeling, are essential components of cancer-associated inflammation. The inflammatory process can be associated with all stages of oncogenesis, from the initial neoplastic transformation to angiogenesis, tissue invasion, and metastasis [6]. 

**Figure 1:**
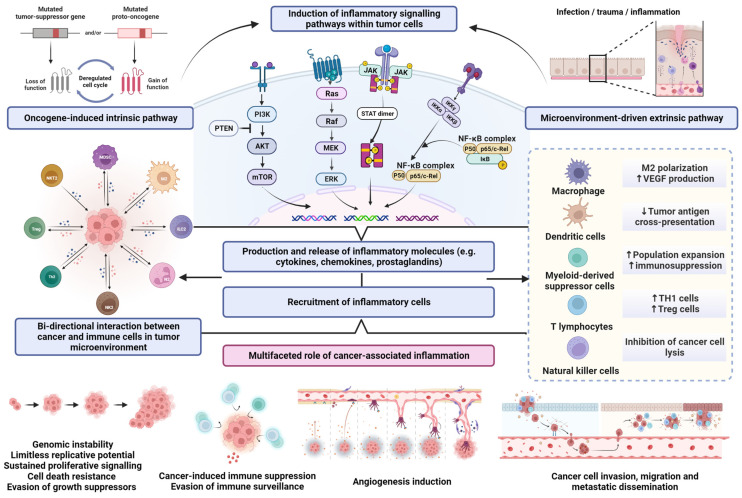
Graphical overview of the multifaceted role of cancer-associated inflammation. Abbreviations: AKT, protein kinase-B; ERK, extracellular-signal-regulated kinase; IKKα, IKKβ, and IKKγ, IκB kinases; JAK, Janus kinase; M2, macrophages type 2; MDSC, myeloid-derived suppressor cells; MAPK, mitogen-activated protein kinases; MEK, mitogen-activated protein kinase; mTOR, mammalian target of rapamycin; N2, type-2 polarized neutrophils; NF-κB, nuclear factor-κB; NKT2, natural killer T cells type 2; p53, tumor protein p53; PI3K, phosphoinositide 3-kinase; PTEN, phosphatase and tensin homolog; RAF, rapidly accelerated fibrosarcoma; RAS, rat sarcoma; STAT, signal transducer and activator of transcription protein; TH1 cells, T helper 1 cells; Treg cells, regulatory T cells; VEGF, vascular endothelial growth factor. The upward pointing arrows indicate increase; the downward pointing arrow indicates decrease.

Acute and properly resolved inflammation is a fundamental response to adverse stimuli and is generally beneficial for the host. In contrast, there is accumulating scientific evidence that chronic, persistent, and deregulated inflammation induced by biological, chemical, or physical factors predisposes risk to various types of malignancies [7]. Sustained inflammatory processes mediate the disruption of cellular homeostasis, facilitate genomic instability, stimulate DNA damage and resistance to apoptosis, suppress antitumor immunity, and promote angiogenesis and the formation of metastatic colonies [8]. Furthermore, inflammatory cells and mediators are present in the majority of neoplastic tissues, even in those where chronic inflammation was not causatively involved in tumorigenesis [9]. Thus, the bidirectional interaction between inflammation and neoplastic transformation and progression includes both oncogene-induced intrinsic pathways and microenvironment-driven extrinsic pathways [10]. In the oncogene-driven pathway, genetic alterations that promote neoplastic transformation also contribute to the development of inflammatory milieu and coordinate the activation and action of specific mediators. Conversely, in the extrinsic pathway, tissue conditions caused by inflammation mediate the continuous phenotypic and functional perturbations within tumor-permissive microenvironments, enabling cell proliferation, neovascularization, and the evasion of immune surveillance, thus supporting malignancy onset and further cancer progression [10,11]. Aberrant tissue homeostasis, coupled with amplified inflammatory signaling, metabolic alterations, and functional adaptations of cancer cells, triggers and invigorates myriad survival pathways, mediating cellular transition towards limitless proliferative potential, the avoidance of immune surveillance mechanisms, and invasiveness [12,13] (Figure 1). 

Pathogenesis of germ cell tumors (GCT) may be influenced by the dysregulation of pro-inflammatory cytokines production and related signaling pathways, accordingly modulating spermatogenesis, causing immunosuppression, increasing the invasiveness of tumor cells, and promoting metastatic dissemination [14]. In penile cancer, two distinct patterns of carcinogenesis are recognized: HPV-dependent and HPV-independent. Chronic inflammatory conditions, such as phimosis, lichen sclerosis, and balanoposthitis, play a crucial role in the HPV-independent pathway [15]. Therefore, experimental and epidemiological research enabling an improved understanding of cancer-associated inflammation represents a scientific platform of immense potential for the development of biomarkers and novel treatment options for these two oncopathologies.

Cancer-associated inflammation and immunosuppression may alter the count of the circulating neutrophils, monocytes, lymphocytes, and platelets. [16]. Neutrophils are crucial elements of inflammation response. The majority of the existing evidence points to neutrophils’ prominent and multifaceted function in tumor development and progression, primarily as inducers of angiogenesis. They promote neoplastic transformation by releasing genotoxic agents and increasing procarcinogenic genetic instability. Furthermore, they facilitate metastatic cascade, tumor cell migration, and invasion [17]. Tumor-mediated granulopoiesis, both in the bone marrow and extramedullary, causes the increased amount of circulating neutrophils and their mobilization [18]. According to several studies, platelets can increase the stability of circulatory tumor cells and accelerate metastatic dissemination by promoting tumor cell adhesion [19]. Lymphocyte depletion, primarily reflected in low CD4+ T cell levels, is a common finding in cancer patients due to the decreased response of adaptive immunity [20]. Based on these findings, multiple inflammatory indices are constructed from these single blood elements in order to objectively evaluate the correlation between immune system, inflammation, and cancer. Easily accessible and cost-effective inflammatory indices may be calculated from routine hematological investigations. In addition, significant changes in other readily accessible inflammatory markers comprising acute phase reactants, primarily as low serum albumin level and increased serum globulins and C-reactive protein (CRP), were observed as a result of cancer-associated inflammation [21,22].

The established multi-level relationship between inflammation and cancer provides a biologic rationale and scientific basis for the implementation of systemic inflammation markers as indicators of oncological outcomes. The prognostic significance of inflammatory indices has been demonstrated in a variety of malignancies, such as breast [23] and colorectal cancer [24] hepatocellular carcinoma [25], melanoma, and a plethora of other solid tumors [26,27]. Furthermore, the prognostic value of various markers of systemic inflammation has been reported in an array of urological cancers, particularly renal cell carcinoma, and bladder and urothelial cancer [28,29]. The aim of this review is to summarize the current scientific data on the prognostic value of systemic inflammatory markers in TC and PC. 

## 2. Materials and Methods

An extensive literature review was carried out by searching the PubMed database for studies published in English language between January 2000 and November 2022. Moreover, a secondary hand-search was undertaken using the reference lists of the identified articles to supplement the initial selection with additional relevant publications. The following Medical Subject Headings thesaurus and free-text terms and abbreviations were applied in the search protocol: “testicular cancer”, “penile cancer”, “systemic inflammation”, “systemic inflammation markers”, “neutrophile-to-lymphocyte ratio” (NLR), “derived neutrophile-to-lymphocyte ratio” (dNLR), “platelet-to-lymphocyte ratio” (PLR), “systemic immune-inflammation index” (SII), “systemic inflammation response index” (SIRI), “lymphocyte-to-monocyte ratio (LMR), albumin-to-globulin ratio (AGR), “C-reactive protein” (CRP), “Glasgow prognostic score” (GPS), and “prognostic nutritional index” (PNI). Unrelated studies, non-English-language articles, articles with unavailable full text, and conference papers were deemed ineligible and therefore excluded from further analysis. To accommodate for the heterogeneity of research approaches and subject complexity, the breadth and depth of the literature search were not limited to a specific study design.

## 3. Systemic Inflammation Markers in Testicular Cancer

### 3.1. Neutrophile-to-Lymphocyte Ratio (NLR)

NLR is calculated by dividing the absolute count of neutrophils by the absolute count of lymphocytes. Among all inflammatory indices, NLR is the most extensively studied as a prognosticator in cancer patients. In a meta-analysis from 2014, which involved over 40,000 patients with solid tumors, NLR greater than the cut-off value of 4 was associated with poor overall survival (OS) [26]. Results from two meta-analyses indicated that elevated pretreatment NLR is a predictor of worse OS, cancer-specific survival (CSS), and recurrence-free survival (RFS) in urological cancers, such as renal cell carcinoma, and bladder and upper-tract urothelial cancer [29,30]. 

The prognostic value of NLR in testicular cancer has been assessed in multiple studies. The majority of these studies used a retrospective design, and the NLR was typically calculated before radical orchiectomy [31]. In the study from 2017 involving 103 patients, Jankovich et al. reported that NLR < 4 was prognostic of non-metastatic disease, while NLR > 4 was statistically significantly associated with a stage of disease higher than T1. The authors concluded that NLR may be considered a valuable prognostic marker for GCT staging [32]. Contrary to these findings, in a cross-sectional study from 2018, which compared testicular cancer patients with a group of cancer-free subjects who underwent varicocelectomy, Gokcen at al. found no significant correlation between NLR and disease stage. Compared with the control group, NLR was significantly higher in the TC cohort [33]. In a study from 2021, Ariman and colleagues retrospectively evaluated the diagnostic and prognostic role of NLR in TC by comparing 152 TC patients with an age-matched control group of 100 healthy male individuals. Testicular cancer patients were allocated into good, intermediate, and poor prognostic groups according to the International Germ Cell Cancer Collaborative Group (IGCCCG) classification. A statistically significant difference was noted for an NLR value between TC patients and the control group, with NLR being significantly lower in the cohort of healthy men. Furthermore, NLR was statistically significantly higher in TC patients with normal serum tumor markers (BHCG, AFP, LDH) compared with the control group. Although the reported NLR was higher in non-seminoma subjects, the difference between those and values found among seminoma patients did not reach the statistical significance threshold. Patients in intermediate IGCCCG and poor prognosis groups had significantly higher NLR compared with good prognosis patients [34]. In another study with a cross-sectional design, similar findings were observed [35]. Results from the aforementioned studies suggest that NLR has diagnostic utility and may complement the well-established tumor markers in clinical practice.

Various studies investigated the prognostic and predictive values of NLR regarding survival and the development of metastatic disease. According to Bolat and colleagues, preoperative NLR is not a reliable predictor of progression-free survival (PFS) and CSS in patients with GCT. Their study was, however, limited by its small sample size (*n* = 53), short follow-up period, and suboptimal AUC value for NLR [36]. In contrast to them, Tan et al. found a significant link between NLR > 3 and low CSS. In their retrospective study involving 160 patients, elevated NLR was also associated with metastatic disease development and retroperitoneal lymph node involvement [37]. In another study from 2019, patients with a pretreatment NLR > 4 had a statistically significantly higher likelihood of developing a more advanced stage of the disease. In addition, NLR displayed significance in predicting low OS and disease progression [38].

Bumbasirevic et al. examined the clinical significance of the intricate relationship between oxidative stress and inflammation in the GCT. Patients with advanced disease (stage II and stage III) exhibited statistically significantly greater levels of NLR and dNLR than stage I patients, providing compelling evidence of the association between a more intensely pronounced inflammatory response and the disease progression. Furthermore, a highly suggestive correlation was reported between the NLR and 8-hydroxydeoxyguanosine, a representative byproduct of oxidative DNA damage [39].

Furthermore, the clinical benefit of NLR was examined in patients with metastatic GCT. Fankhauser and colleagues examined the predictive value of various inflammatory indices, including NLR, in 146 metastatic GCT patients treated with first-line chemotherapy (CHT). In multivariable analysis, NLR > 4.5 was linked with worse OS. The authors suggested integrating IGCCCG classification with systemic inflammatory markers in order to enhance and refine the oncological outcomes prediction [40]. The predictive significance of pre-chemotherapy dNLR was examined by Ribnikar et al. in a retrospective analysis that enrolled 690 GCT patients treated with CHT. While the association between PFS, OS, and dNLR exhibited statistical significance in a univariate analysis, this effect was lost in a multivariate analysis after being adjusted for the IGCCCG classification [41]. Another study published in 2020 evaluated the prognostic value of inflammatory biomarkers in patients with refractory or recurrent GCT undergoing salvage CHT. Based on the receiver operating characteristic curve and multivariable regression analysis featuring inflammatory markers, risk group stratification, patient age, previous treatment, and inferior OS and PFS were correlated with an NLR ≥ 3.3 [42]. The results of discussed studies are summarized in Table 1.

### 3.2. Platelet-to-Lymphocyte Ratio (PLR)

The platelet–lymphocyte ratio is determined by dividing the absolute platelet count by the absolute lymphocyte count from the same blood sample. PLR has received attention in recent years as a potential diagnostic and prognostic marker for a variety of conditions, including multiple cancers, such as hepatocellular and stomach cancer, head and neck squamosal cell carcinoma, non-small-cell lung cancer, renal cell carcinoma, and bladder cancer [43].

In a previously mentioned study from 2018, Gokcen et al. reported that, compared with healthy controls, GCT patients had significantly greater PLR values [33]. Sahin et al.’s cross-sectional study from 2019 employed a comparable methodological approach; however, it included more participants and reported contrasting outcomes. Between the group that underwent a radical orchiectomy for TC (*n* = 120) and the varicocelectomy group (*n* = 171), there was no statistically significant difference in PLR. However, according to paired comparisons of the pathologic primary tumor stage, the PLR of the pT3 group was significantly higher than that of the pT1 and pT2 groups. The pT1 and pT2 groups did not significantly differ in PLR values [44].

According to Herraiz-Raya and colleagues, a PLR > 150 in GCT patients was associated with a greater likelihood of disease progression, advanced stage II and III, and residual disease. Additionally, PLR levels in seminoma patients were statistically significantly higher compared with non-seminoma patients [38]. With a sensitivity of 71% and a specificity of 88%, Imamoglu observed that a PLR > 104 was a significant predictor of advanced disease (stage II and III) in exclusively non-seminoma patients [45].

Peksa et al. analyzed the interconnection between the certain immune checkpoint proteins in TC microenvironments and systemic inflammatory reactions. The calculated PLR cut-off for event predictions was 212. The presence of nodal and distant metastases and an advanced stage of the disease were both associated with elevated PLR. Furthermore, patients with high PLR had statistically significantly better five-year event-free survival in comparison with low-PLR patients (89% vs. 69%, *p* = 0.018). Notably, the combination of high PLR and a low expression of immune checkpoint regulators (V-domain Ig suppressor of T cell activation) in tumor-infiltrating and peritumoral lymphocytes and macrophages was found to be a solitary predictor of relapse and disease progression in multivariate analysis. The findings are consistent with the theory that the clinical behavior of GCT is influenced by a complex interplay between the local tumor immune milieu and the systemic inflammation [46]. 

The predictive value of PLR in the metastatic setting was retrospectively evaluated by Yoshinaga et al. PLR was correlated with poor OS in univariate analysis but was not recognized as a predictor of OS or PFS in multivariate analysis [47]. Similarly, Cursano et al. observed that PLR was not an independent predictor for OS in patients with recurrent GCT, even though PLR > 170 was linked with a favorable response to CHT [42]. An overview of the discussed studies is presented in Table 2.

### 3.3. Systemic Immune-Inflammation Index (SII)

The systemic immune-inflammation index is a complex and potentially more robust and objective biomarker compared with NLR and PLR due to the integration of three different types of peripheral blood cells. SII is determined using the formula PxN/L, where P, N, and L represent the absolute counts of platelets, neutrophils, and lymphocytes in peripheral blood, respectively. According to several meta-analyses, SII may serve as a potent predictor of oncological outcomes in solid tumors, including different urological malignancies [28,48].

Kalavska et al. examined the relationship between SII level and innate and adaptive immune system response in 51 pre-CHT TC patients. Univariate analysis has shown that, among various innate immune cell subpopulations, SII level < 1003 was statistically significantly related to a decreased percentage of neutrophils and natural killer (NK) cells, along with elevated percentages of eosinophils, basophils, dendritic, and plasmacytoid dendritic cells. However, only the neutrophils percentage was confirmed as an independent predictor of SII in the multivariate analysis. Furthermore, a flow-cytometry-based immunophenotyping analysis of adaptive immune cell populations revealed that the low-SII patient group (SII < 1003) had significantly elevated percentages of lymphocytes, T cell lymphocytes, and cytotoxic T lymphocytes compared with the high-SII group. Nevertheless, in multivariate analysis, only CD3+ T cells were found to be an independent predictor of SII level in TC patients. Detected changes in innate and adaptive immune cell populations can be a significant indicator of tumor-induced immunosuppression [49].

Multiple authors assessed the prognostic and predictive role of pre-CHT SII in GCT patients. In a retrospective translational study from 2018, Chovanec et al. detected that elevated SII was significantly associated with multiple adverse clinicopathological parameters, such as poor and intermediate IGCCCG risk groups, bulky retroperitoneal lymphadenopathy, and elevated serum tumor markers. Additionally, when compared with the low-SII group, patients in the high-SII group had significantly worse OS and PFS, thus underscoring the predictive relevance of the SII. SII was a significant OS predictor in the multivariate analysis, independent of the IGCCCG risk category. The expression of programmed death ligand 1 (PD-L1), an immune checkpoint protein, in tumor-infiltrating lymphocytes and tumor cells did not significantly correlate with SII levels. Based on the integrated prognostic impacts of the PD-L1 and SII, three prognostic group were developed. Patients with low PD-L1 expression and high SII had a worse prognosis than those in other groups [50]. In another retrospectively designed study involving 112 participants, Imamoglu and colleagues stated that median SII values between stage I and advanced-stage seminoma patients were statistically significantly different. SII > 672 was a predictor of the advanced stage, with a sensitivity and specificity of 59% and 75%, respectively [45]. Similar results were obtained by Bumbasirevic et al. in a prospective study from 2022. Median SII levels were significantly different in stage I patients compared with stage II and III patients (533.33 vs. 824.26, *p* < 0.001). SII was associated with metastatic disease development at the cut-off of 683.21 (AUC 0.714), with a reported sensitivity and specificity of 66.10% and 70.37%, respectively. Additionally, the authors provided interesting insight into the complex interaction between cancer-induced inflammation and oxidative DNA damage by identifying a highly suggestive correlation between SII and modified nucleoside 8-hydroxydeoxyguanosine (8-OHdG) [39]. In the current literature, findings regarding the predictive value of the SII in patients with metastatic GCT remained inconclusive. While Fankhauser [40] and Cursano [42] reported that elevated SII is significantly associated with shorter OS, Yoshinaga [47] did not confirm these results. The results of the discussed studies are summarized in Table 3.

### 3.4. Lymphocyte-to-Monocyte Ratio (LMR)

Another systemic inflammatory biomarker based on common hematological elements is the lymphocyte-to-monocyte ratio, which is, in comparison with others, less extensively studied [45]. Results from several meta-analyses indicate that decreased levels of LMR can be associated with worse OS, PFS, and CSS [51,52]. In the meta-analysis from 2019, which examined the prognostic significance of LMR in renal cell carcinoma, bladder cancer, and upper-tract urothelial carcinoma, low LMR was correlated with poor OS, advanced tumor stage and grade, and the occurrence of lymph node metastases [53].

The prognostic role of LMR in GCT patients was predominantly assessed prior to radical orchiectomy regarding disease progression and metastases development. The majority of research that has been published on this subject consistently demonstrated that stage I patients have statistically significantly greater values of LMR than patients with advanced-stage (stage II + III) disease [38,39,45,46,54]. In an observational study from 2020, Olcucu et al. reported that patients with nodal and distant metastases had significantly lower median LMR compared with non-metastatic GCT patients [54]. Similar findings were observed by Peksa et al. in a retrospective analysis from 2021, while Bumbasirevic and colleagues found that preoperative LMR, at the cut-off of 4.14 (AUC 0.730, *p* = 0.001), predicted metastatic disease occurrence, with a sensitivity and specificity of 50.84% and 85.18%, respectively [39,46]. Likewise, lower LMR can be associated with a higher incidence of residual disease [38]. There was no evidence of a significant relationship between LMR and OS in GCT patients in the published literature, which may be partially explained by the relatively small patient cohorts [46,54].

### 3.5. C-Reactive Protein and Albumin-Related Markers

CRP is a quintessential regulatory protein of the acute phase response, whose blood levels reflect the degree of the immune system reaction to tissue damage and resulting inflammation. An improved understanding of its dynamic nature and capacity to undergo non-proteolytic conformational modification yielding two distinct isomeric forms, with unique inflammatory-response-related activities, extended substantially the appreciation of its role in cancer-associated inflammation [55]. Pentameric CRP (pCRP), an isoform of CRP that is detected and quantified in blood tests, exhibits relatively modest pro-inflammatory activity. The binding of pCRP to cell membranes instigates a pentamer dissociation to monomeric subunits (mCRP), with potent pro-inflammatory bioactivity. While the mCRP is mostly activated in the early acute phase response, pCRP accumulation in the blood is deemed indicative for ongoing, low-level inflammation, which can be a marker of an unresolved and progressive disease, such as cancer [56].

There are limited data concerning CRP as a biomarker in TC patients. The correlation between routinely assessed markers of inflammation, including CRP, and the risk of TC and PC was explored within the Swedish Apolipoprotein-related Mortality Risk (AMORIS) study. The study had a prospective cohort design and involved 202,717 participants. After a median follow-up of 20.3 years, TC was diagnosed in 125 patients. A statistically significant association between CRP levels and the risk of TC was not observed [57]. The previously mentioned study from 2022 that examined the interaction between pre-orchiectomy inflammation and redox biomarkers in GCT patients found a significant correlation between CPR levels and thiol group levels, suggesting a potential link between inflammation and oxidative-stress-induced protein damage. Additionally, CRP values correlated with maximal tumor dimensions and higher stages of disease [39]. Only one study investigated the predictive utility of CRP in metastatic GCT patients. Although elevated CRP was associated with shorter OS according to univariate analysis, the multivariate analysis did not confirm these findings [40].

Hypoalbuminemia is commonly associated with chronic inflammatory processes. It is proposed that interleukin-1 produced by monocytes is a crucial mediator of reduced albumin synthesis in inflammation [58]. Contrary to albumins, long-term inflammatory disorders, including cancer, diabetes, or chronic liver diseases, are inducers of elevated serum globulin synthesis [21]. The albumin-to-globulin ratio (AGR) was designed to provide a more objective assessment of the interaction between serum proteins and chronic inflammation. While low AGR was found to be a reliable indicator of poor prognosis in gastric, esophageal, and lung cancer, similar studies on urological malignancies revealed conflicting results [59,60,61,62,63].

The prognostic relevance of AGR in testicular cancer was examined only in three studies. In a retrospective analysis of 115 patients, Guner et al. observed that an AGR < 1.47 was a significant predictor of retroperitoneal lymph node and distant metastases. Kaplan–Meier analysis revealed a significant correlation between an AGR < 1.47 and poor OS [64]. By defining the cut-off value of AGR at 1.53, Bumbasirevic et al. also regarded the significance of this biomarker as prognostic of metastatic disease. Additionally, it was found that stage I GCT patients had significantly higher AGR values in comparison with stage II and III patients (1.84 vs. 1.62, *p* = 0.009). Parallel to CRP, a significant association was detected between AGR and the levels of oxidative-stress-induced protein damage byproducts [39]. In a retrospective study from 2020 that included 66 pre-CHT GCT patients, Yoshinaga and colleagues reported that AGR was a significant predictor of OS. Alongside AGR, the authors evaluated the prognostic value of two additional inflammation- and albumin-based biomarkers, namely the Glasgow prognostic score (GPS) and the prognostic nutritional index (PNI). GPS, a biomarker based on albumin and CRP levels, was a significant predictor of OS and PFS in both univariate and multivariate analyses, in contrast to PNI, an index calculated from serum albumin levels and absolute lymphocyte count, which was only linked with PFS in univariate analysis [47].

## 4. Systemic Inflammation Markers in Penile Cancer

### 4.1. Neutrophile-to-Lymphocyte Ratio (NLR)

Similar to TC, NLR is the most extensively investigated marker of systemic inflammation in patients with PC. The majority of studies used a retrospective design, with a variable number of patients (ranging from 39 to 228). NLR was predominantly assessed prior to inguinal lymphadenectomy (ILND). Kasuga et al. examined the prognostic value of NLR in 41 patients undergoing total penectomy for penile squamous cell carcinoma (PSCC). Patients with an NLR > 2.82 had significantly inferior OS and CSS compared with the low-NLR patient group. Furthermore, elevated NLR significantly correlated with the presence of inguinal lymph node metastases [65]. Another study with a rather limited number of patients (*n* = 39) and an equivalent cut-off value of 2.8 found a significant correlation between high pretreatment NLR and poor CSS [66]. In a retrospective analysis of 84 PSCC patients who underwent ILND, Azizi and colleagues found that patients with an NLR > 3 were more likely to have a higher stage of disease, pathological lymph node involvement (pN+), and extranodal extension (ENE) compared with patients with an NLR < 3. On univariate analysis, higher NLR was associated with poor OS, CSS, and recurrence-free survival (RFS). Multivariate analysis, however, revealed that NLR had an independent effect only on OS. Along with lymphovascular invasion (LVI) and clinically positive lymph nodes, elevated NLR was a predictor of pN+ disease (odds ratio (OR) = 3.75; 95% confidence interval (CI): 1.30–10.81, *p* = 0.014) [67]. An NLR > 2.94 was an independent predictor of pN+ in another retrospective cohort study of 225 patients treated with ILND. Elevated NLR was significantly correlated with worse OS and PFS, but only by conducting a univariate analysis [68]. Jindal et al. reported concordant results regarding the association between NLR and pN+ disease and survival outcomes [69]. Li et al. investigated the prognostic significance of NLR in 228 patients treated with bilateral ILND for PSCC. According to the authors, NLR exhibited the highest prognostic accuracy among several inflammatory biomarkers. The significant correlation between NLR and CSS was demonstrated in both univariate and multivariate analyses [70]. An overview of the discussed studies is presented in Table 4.

### 4.2. Leukocytes and Platelets-Related Markers

The aforementioned study by Li and colleagues provided the initial evaluation of the prognostic value of PLR in PC patients. PLR correlated with CSS rate in univariate analysis, with a cut-off value of 169. Among other inflammatory indices, PLR had the best predictive accuracy after NLR (bootstrap C-index 0.602) [70]. Hu et al. revealed a significant association between PLR value and OS and PFS. Additionally, PLR was found to be an independent predictor of pathological N stage (HR = 2.478, 95% CI: 1.365–4.497, *p* = 0.003) [68]. In accordance with previous publications, Song et al. found that patients with elevated PLR values had significantly inferior OS compared with the low-PLR patient group [71]. Wu et al. analyzed the predictive utility of PLR for the pathological outcomes of ILND. PLR was found to be the most accurate predictor of inguinal lymph node metastases and lymph node ENE among various laboratory and pathological variables [72].

In contrast to TC, where SII was moderately investigated, only one study examined the predictive significance of SII in patients with penile cancer. Song and colleagues retrospectively assessed the SII values in 123 patients prior to partial or total penectomy. Patients treated with partial penectomy had significantly lower SII levels compared with those in the total penectomy group (*p* = 0.027). The estimated cut-off level for SII in the study was 636.99. There was a statistically significant difference regarding the median OS between the high and low SII groups of patients (10.5 months vs. 128 months, *p* = 0.01) [71].

Studies that assessed the prognostic and predictive values of LMR in patients with PC revealed conflicting findings. While studies from 2017 and 2021 reported that LMR values below the estimated cut-off points were associated with inferior CSS, Hu and colleagues stated that patients with decreased LMR values had significantly better OS compared with those with high LMR values (68, 70, 71). Hu and Jindal, however, concurred in their findings that a lower LMR was a predictor of pathological inguinal lymph node involvement [68,69].

### 4.3. C-Reactive Protein and Albumin-Related Markers

A possible prognostic role of CRP in PC was investigated by several studies. The first study, conducted by Steffens et al., found a strong correlation between increased circulating levels of CRP (>15 mg/l), the advanced stage of disease (≥pT2), and the presence of inguinal lymphadenopathy at the time of diagnosis. Patients with elevated CRP levels had a significantly inferior 5-year CCS rate compared with those with lower levels of CRP (38.9% vs. 84.3%, *p* = 0.001). According to multivariate analyses, elevated CRP was independently associated with poor clinical outcomes in PC patients (HR: 3.34, 95% CI: 1.04–10.7, *p* = 0.043) [73]. High preoperative CRP (>20 mg/L) was a predictor of inguinal lymph node metastases in another retrospective study [74]. Both studies, however, were limited by a small number of patients.

The above-mentioned AMORIS study explored the potential association between the development of TC and PC and commonly measured inflammatory biomarkers, such as CRP, albumin, and globulin. Similar to TC, a statistically significant correlation between these serum markers and the risk of PC was not demonstrated [57]. 

The predictive value of the albumin-related markers of inflammation in PC patients was examined only in one recent study from 2022. In this retrospective analysis that included 123 patients undergoing penectomy, patients with lower PNI values had statistically significantly worse OS compared with the high PNI group (100.4 months vs. 135.8 months, *p* < 0.001). Similar findings were observed in the comparison of the low AGR and increased AGR group (75.7 months vs. 128.2 months, *p* > 0.001). Both PNI and AGR were identified as independent predictors of OS in a multivariate analysis [71]. 

## 5. Systemic Inflammation Markers Limitations

Several inherent drawbacks of systemic inflammation markers should be acknowledged, as they may hamper validity and clinical utility. These indicators are susceptible to variations driven by circadian intra-individual fluctuations, miscellaneous physiological or pathological processes, non-cancer-related conditions, prior medical procedures or administered therapeutic agents, and sampling circumstances. Specimen harvesting in a controlled clinical environment under consistent and standardized conditions performed in conjunction with comprehensive patient assessment should be applied to address these concerns and manage potential performance repercussions [39]. Furthermore, it is noteworthy that, despite, reasonable congruity across the currently available research corpus, there is a lack of universally accepted sensitivity and specificity rates and threshold values for these indicators. The challenge in reaching a scientific and professional concord on this topic, including the endorsement of specific quantitative standards for inflammatory classifiers as prognostic markers in TC and PC, is attributable to the non-negligible heterogeneity in the published data regarding the employed methodological approach, observed clinical determinants, reporting quality, and the analyzed end-point variables.

## 6. Conclusions

Systemic inflammation markers are inexpensive and widely available indices with the potential to complement the established biomarkers and clinicopathological parameters in patients diagnosed with TC and PC. The implementation of these biomarkers in clinical practice may improve current risk-stratification protocols and decision-making processes and enhance personalized treatment. The accumulated scientific evidence addressed in the present review underscores NLR as the most prominent inflammatory index with noteworthy prognostic relevance for TC. Other biomarkers, particularly SII and PLR, have also been credited as valuable prognostic indicators, although they require further evaluation, primarily via prospective, multicenter studies involving larger numbers of patients and meta-analysis. In comparison with TC, the published literature on systemic inflammation biomarkers in PC is quite scarce. Although the available research evidence points to NLR as a potentially useful prognosticator for this urological malignancy as well, additional research is warranted to explore this and other biomarkers. 

Future research endeavors in this field should address certain limitations and pitfalls. The considerable variations in biomarker cut-off values between studies impede the establishment of a professional consensus on this matter and hinder the routine application of these indices in standard clinical practice. To overcome this challenge, there is a need for adequately powered studies with a standardized design featuring high scientific rigor, a harmonized methodological approach, proper control mechanisms for confounders, and consistent end-point variables. Targeted research on the prognostic value of inflammatory biomarkers in specific subgroups of TC and PC patients, such as seminoma vs. non-seminoma patients, recurrent TC patients, clinically node-negative PC patients, and pre-CHT metastatic PC patients should be encouraged as well. Lastly, an advanced investigation into the complex interconnections between systemic inflammation and carcinogenesis, accounting for the intricate and multifarious role of the tumor microenvironment, may improve the general understanding of tumor biology, clinical behavior, and treatment response, thus enhancing overall therapeutic care in these patient populations.

## Figures and Tables

**Table 1:** Prognostic importance of NLR in testicular cancer patients.

Author (Year)	Reference	Patients (*n*)	Study Design	Cut-Off Value	Main Findings
Yuksel (2016)	[35]	36	Cross-sectional	-	NLR was significantly elevated in TC patients compared with the control group
Jankovich (2017)	[32]	103	Retrospective cohort	4.0	NLR < 4 was a predictor of non-metastatic disease NLR > 4 was associated with stage > T1
Bolat (2017)	[36]	53	Retrospective cohort	3.55 for PFS 3.0 for CSS	Preoperative NLR was not a reliable predictor of PFS and CSS
Gokcen (2018)	[33]	39	Cross-sectional	-	NLR was significantly elevated in TC patients compared with the control group
Fankhauser (2018)	[40]	146	Retrospective cohort	4.5	NLR > 4.5 was associated with poor OS in metastatic GCT patients undergoing first-line CHT
Tan (2019)	[37]	160	Retrospective cohort	3.0	NLR > 3 was a significant predictor of poor CSS, lymph node involvement, and metastatic disease
Herraiz-Raya (2019)	[38]	164	Retrospective cohort	4.0	NLR > 4 was associated with advanced stage of disease and poor OS
Cursano (2020)	[42]	146	Retrospective cohort	4.5	NLR > 4.5 was significantly correlated with poor OS in metastatic GCT patients
Ariman (2021)	[34]	152	Cross-sectional	-	NLR was significantly elevated in TC patients compared with the control group Patients with intermediate and poor IGCCCG prognosis had significantly elevated NLR compared with patients with good IGCCCG prognosis
Bumbasirevic (2022)	[39]	88	Prospective cohort	2.685	NLR > 2.685 was associated with metastatic disease NLR was significantly increased in Stage II and III patients compared with Stage I patients NLR was associated with 8-hydroxydeoxyguanosine, a representative byproduct of oxidative DNA damage

NLR, neutrophile-to-lymphocyte ratio; TC, testicular cancer; PFS, progression-free survival; CSS, cancer-specific survival; CHT, chemotherapy; OS, overall survival; IGCCCG, International Germ Cell Cancer Collaborative Group.

**Table 2:** Prognostic importance of PLR in testicular cancer patients.

Author (Year)	Reference	Patients (*n*)	Study Design	Cut-Off Value	Main Findings
Gokcen (2018)	[33]	39	Cross-sectional	-	PLR was significantly elevated in TC patients compared with the control group
Sahin (2019)	[44]	120	Cross-sectional	-	There was no statistically significant difference in PLR value between TC patients and the control group
Imamoglu (2019)	[45]	112	Retrospective cohort	104	PLR > 104 was significantly associated with advanced disease (Stage II + III) in non-seminoma patients
Herraiz-Raya (2019)	[38]	164	Retrospective cohort	150	PLR > 150 was associated with a greater likelihood of disease progression, advanced disease (stage II and III), and residual disease PLR values in seminoma patients were statistically significantly higher compared with non-seminoma patients
Yoshinaga (2020)	[47]	63	Retrospective cohort	-	High PLR was linked with poor OS only in univariate analysis
Cursano (2020)	[42]	62	Retrospective cohort	170	PLR > 170 was associated with a favorable response to CHT
Peksa (2021)	[46]	180	Retrospective cohort	212	PLR > 212 was associated with an advanced stage of disease and the presence of nodal and distant metastasis PLR > 212 was correlated with poor EFS

PLR, platelet-to-lymphocyte ratio; TC, testicular cancer; EFS, event-free survival; CHT, chemotherapy; OS, overall survival.

**Table 3:** Prognostic importance of SII in testicular cancer patients.

Author (Year)	Reference	Patients (*n*)	Study Design	Cut-Off Value	Main Findings
Chovanec (2018)	[50]	171	Retrospective translational	1003	SII > 1003 highly correlated with intermediate and poor IGCCCG risk groups, bulky retroperitoneal lymphadenopathy, and elevated tumor markers SII > 1003 was associated with poor OS The combination of low PD-L1 expression and elevated SII correlated with poor prognosis
Fankhauser (2018)	[40]	146	Retrospective cohort	1428	SII > 1428 was significantly associated with poor OS
Imamoglu (2019)	[45]	112	Retrospective cohort	672	SII > 672 was associated with an advanced disease stage
Cursano (2020)	[42]	62	Retrospective cohort	844	SII > 844 highly correlated with worse OS and PFS SII > 844 was associated with poor response to CHT
Yoshinaga (2020)	[47]	63	Retrospective cohort	-	SII was not associated with OS
Bumbasirevic (2022)	[39]	88	Prospective cohort	683.21	Median SII values were significantly lower in Stage I patients compared with Stage II and III patients SII > 683.21 was associated of metastatic disease development SII was associated with 8-hydroxydeoxyguanosine, a representative byproduct of oxidative DNA damage
Kalavska (2022)	[49]	51	Retrospective cohort	1003	SII > 1003 was associated with an increased percentage of neutrophils and decreased percentage of lymphocytes

SII, systemic immune-inflammation index; IGCCCG, International Germ Cell Cancer Collaborative Group; OS, overall survival; PFS, progression-free survival; CHT, chemotherapy; PD-L1, programmed death-ligand 1.

**Table 4:** Prognostic importance of NLR in penile cancer patients.

Author (Year)	Reference	Patients (*n*)	Study Design	Cut-Off Value	Main Findings
Kasuga (2016)	[65]	41	Retrospective cohort	2.82	NLR > 2.82 highly correlated with poor OS and CSS and pN+ disease
Tan (2017)	[66]	39	Retrospective cohort	2.8	NLR > 2.8 was associated with poor CSS
Azizi (2019)	[67]	84	Retrospective cohort	3.0	NLR > 3 was significantly associated with a higher stage of disease, pN+ disease, ENE and poor OS
Li (2020)	[70]	228	Retrospective cohort	-	Elevated NLR highly correlated with worse CSS
Hu (2020)	[68]	225	Retrospective cohort	2.94	NLR > 2.94 was associated with pN+ disease NLR > 2.94 highly correlated with poor OS and PFS, but only in univariate analysis
Jindal (2021)	[69]	69	Retrospective cohort	3.0	NLR > 3 was associated with pN+ disease and poor CSS

NLR, neutrophile-to-lymphocyte ratio; OS, overall survival; CSS, cancer-specific survival; pN+ disease, pathological inguinal lymph node involvement; ENE, extranodal extension; PFS, progression-free survival.
